# Handy and highly efficient oxidation of benzylic alcohols to the benzaldehyde derivatives using heterogeneous Pd/AlO(OH) nanoparticles in solvent-free conditions

**DOI:** 10.1038/s41598-020-62695-4

**Published:** 2020-03-31

**Authors:** Haydar Goksu, Fatih Sen

**Affiliations:** 1 Kaynasli Vocational College, Duzce University, Düzce, 81900 Turkey; 2 Sen Research Group, Department of Biochemistry, Dumlupinar University, 43100 Kütahya, Turkey

**Keywords:** Biochemistry, Biochemistry, Catalysis, Catalysis

## Abstract

The selective oxidation of benzylic alcohols was performed by using commercially available aluminum oxy-hydroxide-supported palladium (Pd/AlO(OH)) nanoparticles (0.5 wt.% Pd, about 3 nm size) under mild conditions. The oxidation method comprises the oxidation of benzyl alcohols catalyzed by aluminum oxy-hydroxide-supported palladium under ultrasonic and solvent-free conditions and a continuous stream of O_2_. The characterization of aluminum oxy-hydroxide-supported palladium nanocatalyst was conducted by several advanced analytical techniques including scanning electron microscope (SEM), transmission electron microscope (TEM), X-ray diffraction (XRD), and elemental analysis by ICP-OES. The oxidation of a variety of benzyl alcohol compounds were tested by the aluminum oxy-hydroxide-supported palladium nanoparticles, and all expected oxidation products were obtained by the high conversion yields within 3 hours. The reaction progress was monitored by TLC (Thin-layer chromatography), and the yields of the products were determined by ^1^H-NMR and ^13^C NMR analysis.

## Introduction

**Figure 1:**
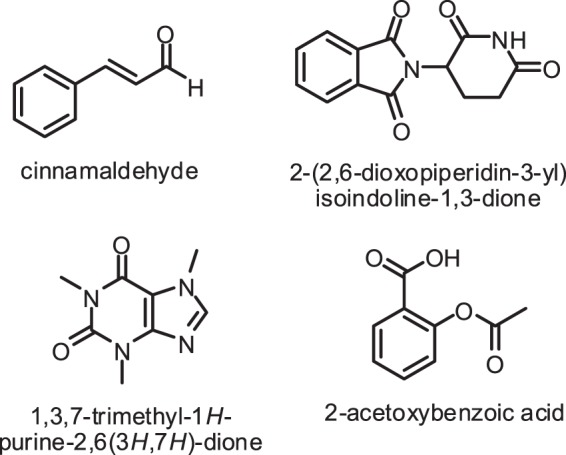
Biologically active materials.

The carbonyl compounds obtained by the oxidation of alcohol compounds are important intermediates used in the production of new molecules in both chemistry and industry. Therefore, oxidation reactions are especially important for organic chemists. For instance, cinnamaldehyde^1^, 2-(2,6-dioxopiperidin-3-yl) isoindoline-1,3-dione^2^, 1,3,7-trimethyl-1H-purine-2,6(3 H,7 H)-dione^3^ and 2-acetoxybenzoic acid^4^ are biologically active materials (Fig. 1).

The permanganate, chromium trioxide, dichromate, chromic acid are the primary oxidants used for the oxidation of alcohols. These oxidants are both expensive and toxic, therefore, not suitable for use^5^. It also causes oxidation of primary alcohols to carboxylic acids by giving uncontrolled oxidation reactions^6^. Of course, the solvent in the reaction medium is important for advanced oxidation or the absence of solvent^7^.

**Figure 2:**
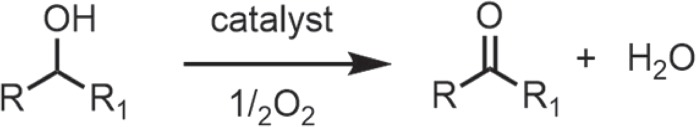
Oxidation of alcohol compounds to carbonyl compounds.

However, in recent years, molecular oxygen is used as the primary oxidant, considering both environmental and economic conditions. By the use of molecular oxygen, the alcohol compounds are oxidized to carbonyl compounds, and the water molecule is formed in the medium (Fig. 2). Of course, the molecular oxygen is used in conjunction with different metal catalysts such as CuMn_2_^8^, [VO(TPPABr)] CBr_3_)^9^, Cu (II)-Ligand^10^, Pd^11^, and CuSO_4_^12^.

Aluminum oxy-hydroxide supported palladium nanoparticles are, unfortunately, a commercial catalyst with limited usage as a heterogeneous catalyst. However, in recent years there has been an increase in the number of publications with the catalyst (alkylation^13^, reductions,^13^ dynamic kinetic resolution,^13^ hydrogenation^14,15^). On the other hand, aluminum oxy-hydroxide-supported palladium nanoparticles were used in the selective degradation of azide and nitro in the dehalogenation reactions, in the Suzuki Cross-Coupling reactions and in the Knoevenagel condensation reactions by our group for the first time^16^. This catalyst may be preferred because it is stable under different reaction conditions.

Pd/AlO (OH) catalyst, which has a commercial value, is stable, does not decompose under room conditions or external effects, is reused and recovered by a simple method is the preferred reason of this catalyst. In addition, the support (AlO(OH)) used in the catalyst has a basic character, which provides an auxiliary role for the KOH used in this study. Thus, it can be employed for the oxidation of benzylic alcohols to benzaldehyde derivatives.

Herein, a well-known easy route for the oxidation of benzylic alcohol compounds to aldehyde derivatives under ambient conditions is reported. The oxidation method used in this study comprises the oxidation of primary alcohols catalyzed by aluminum oxy-hydroxide-supported palladium nanoparticles under ultrasonic and solvent-free conditions. Various benzylic alcohol compounds were tested with aluminum oxy-hydroxide supported palladium nanoparticles, obtained with recovery’s efficiency of up to 99% of all expected oxidation products over 180 min. reaction time.

The reaction is carried out in the presence of a commercially available heterogeneous catalyst. Furthermore, although the synthesis of benzaldehyde derivatives from benzylic alcohol derivatives is frequently encountered in the literature, reactions are carried out under solvent-free conditions. Furthermore, the formation of carboxylic acid derivatives can be observed in such oxidation reactions. However, in this study, we were able to keep the oxidation at the aldehyde step with high yields. The repeated use of the catalyst is indicative of an economical method.

## Results and Discussion

**Figure 3:**
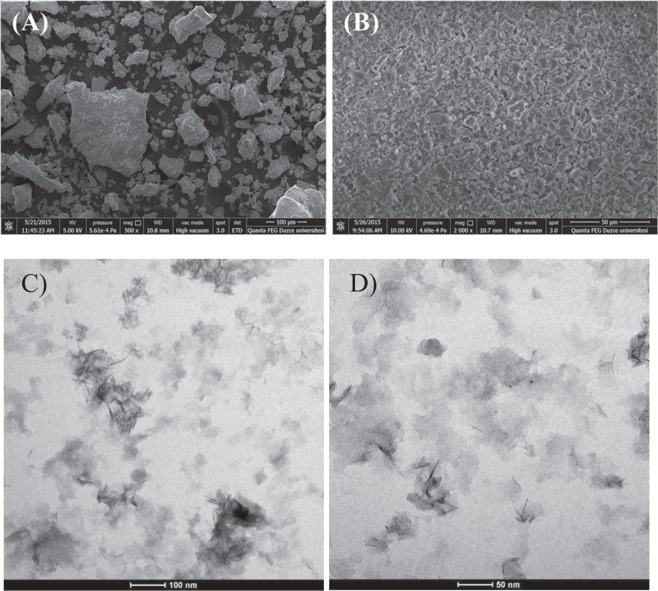
SEM images of Aluminum oxy-hydroxide-supported palladium nanoparticles (**A**) before the reaction; (**B**) after reusing five times. TEM images of Pd/AlO(OH) (**C**) 100 nm scale (**D**) 50 nm scale.

SEM, TEM and XRD analyses of aluminum oxy-hydroxide-supported palladium nanoparticles before and after reaction were performed. Figure 3(a,b) show that the aluminum oxy-hydroxide-supported palladium nanoparticles have a nanocrystalline Boehmite structure with an irregular spread. In Fig. 3(b), aluminum oxide-hydroxide supported palladium nanoparticles were found to be agglomerated on the surface of the catalyst after repeated use of five times. The composition of the catalyst obtained consists of palladium, aluminum, oxygen, and carbon (Figs. S1 and S2). The percentage of carbon from the air should not be ignored.

TEM images, before and after using in reactions, were illustrated in Fig. 3(c,d) and Fig. S4. TEM images revealed that Pd/AlO(OH) nanocatalyst has a sheet-like transparent structure. In some parts, Pd nanoparticles can be clearly seen (marked with arrows), before and after the experimental studies Pd nanoparticles remain entrapped in AlO(OH) structure which is evidence for the stability of the catalyst. TEM images also showed that the catalyst has a mean particle size of about 3 nm, the small crystal size of Pd nanoparticles are highly affecting the catalytic activity^17^.

The XRD pattern of Pd/AlO(OH) nanocatalyst is shown in Fig. S3. The peaks appeared at 2Ɵ degrees of 13.0°, 27.7°, 64.7°, and 71.8° indicated the presence of (020), (120), (002) and (251) planes of AlO(OH), respectively. The peaks show that the structure of the palladium on the surface of the AlO(OH) has a face-centered cubic (fcc) structure. This structure has been proven by previously published studies^18^. Moreover, the additional peaks observed at 38.2°, 49.1°, 85.5 correspond to (111), (200), and (222) planes of cubic structured Pd nanoparticles, respectively. The results indicate that Palladium nanoparticles were distributed onto AlO(OH) support material.

The catalytic activity of the aluminum oxy-hydroxide-supported palladium nanoparticles was studied for the selective oxidation of benzyl alcohols to benzaldehyde in the presence of oxygen gas, in the presence and absence of a base (Table 1). Firstly, although the reaction lasted for 6 hours, there was no trace of the product in the absence of a base. The yield of benzaldehyde obtained after 6 h was found to be only 12% and 65% in the presence of 1 mmol of K_2_CO_3_ and KOH, respectively (Table 1, entries 2, 3). The reactions were carried out in solvent-free conditions. The addition of 1.5 mmol of KOH showed a serious increase in the yield (Table 1, entry 4). The catalyst amount was reduced to half, and the benzaldehyde was obtained by high yields within 3 hours, using 1.5 mmol of KOH (Table 1, entry 5). However, there was no benzaldehyde formation in the absence of a catalyst (Table 1, entry 6). Sonication conditions (50 Hz, 100 W, 40 °C) were used to accelerate the reaction. The yields were found to be much lower when the reactions were carried out at room temperature (without ultrasonic conditions) (Table 1, entry 7). On the other hand, in the absence of conditions, high temperatures are needed to increase efficiency. Since benzyl alcohol derivatives are usually in liquid form, a solution medium is formed. In this case, the solubility of KOH, a strong base, increases with the effect of sound waves under ultrasonic conditions and facilitates the transfer of the proton in the benzylic position onto the palladium. Because the reaction does not take place in the absence of a base. The strength of the base also affects the reaction efficiency. All trials in Table 1 were performed at least 3 times.

**Table 1:** Optimization experiments for the oxidation of benzyl alcohol to benzaldehyde.


Entry	Catalyst, mg	Base	Time (h)	Yield^*b*^ (%)
1	50	—	6	Trace
2	50	K_2_CO_3_ (1 mmol)	6	12 ± 1
3	50	KOH (1 mmol)	6	65 ± 2
4	50	KOH (1.5 mmol)	6	>99 ± 2
**5**	**25**	**KOH (1.5 mmol)**	**3**	**>99** ± 2
6	–	KOH (1.5 mmol)	3	Trace
7^*c*^	25	KOH (1.5 mmol)	3	30 ± 3

^a^Reaction Conditions: 1 mmol substrate, Aluminum oxy-hydroxide-supported palladium nanoparticles (0.5 wt % Pd), a continuous stream of O_2_. ^b^Determined by ^1^H NMR analysis. ^c^At room temperature.

The different bases are also used in the literature, but we have accelerated the breakage of the benzylic proton using a base such as KOH. In addition, the oxidation reaction was accelerated by using sound waves under ultrasonic conditions. The oxidation of benzyl alcohols was carried out, especially in a solvent-free environment, with the activity of the catalyst. The solvent could be used in the reactions. However, an organic solvent (DMSO, THF, Toluene, MeOH, EtOH, etc.) would be preferred. Most of these preferred organic solvents are carcinogenic and pose serious environmental hazards. It causes an accumulation of important problems by mixing with water or air as waste. Also, considering the economics of the reaction, solvent-free reaction conditions are quite economical.

Compared with other studies on the oxidation of benzylic alcohols to benzaldehyde derivatives, the catalytic activity of aluminum oxide-hydroxide supported palladium nanoparticle was found to be much higher (see Table S1). The use of an aluminum oxy-hydroxide-supported palladium nanoparticles may be preferred on account of product diversity, product yield, reaction temperature and time. Eventually, as shown in Table S1, the performance of aluminum oxy-hydroxide-supported palladium nanoparticles has been compared with the other catalysts in literature^8,9,12,19–23^ for the model reaction, and it was found that the aluminum oxy-hydroxide-supported palladium nanoparticles have shown the best performance compared to the others in terms of time, temperature and reaction yield. In literature reviews, it is seen that metal alone or support alone is not sufficient. The two are whole and have a holistic effect. This is demonstrated once again by the preferred catalyst and the method developed in this study. Particularly the reaction temperature and time have been advantageous with the catalyst used.

**Figure 4:**
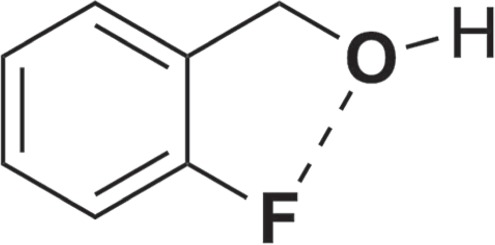
The coordination of the fluorine atom with the alcohol group.

Table 2 summarizes the results obtained from aluminum oxy-hydroxide-supported palladium nanoparticles catalyzed oxidation reactions. Under ultrasonic conditions, benzyl alcohol compounds tested in 180 min without solvent were oxidized to benzaldehyde derivatives in high yields. The 2-fluorobenzyl alcohol (1) has a fluorine atom in the ortho position. The coordination of the fluorine atom with the alcohol group ensures that the reaction is complete with low yields (75%) (Fig. 4). 4-fluorobenzaldehyde (4), 4-bromobenzaldehyde (6), 3,4-dichlorobenzaldehyde (8) were obtained with yields of more than 96% (Table 2, entries 2–4).

**Table 2:** Aluminum oxy-hydroxide-supported palladium nanoparticles catalyzed the oxidation of various benzyl alcohol compounds.


Entry	Substrate	Product	Conv^*b*^/Sel^*c*^/Yield^*d*^%	Entry	Substrate	Product	Conv^*b*^/Sel^*c*^/Yield^*d*^%
1	(1)	(2)	75/100/75 ± 2	8	(15)	(16)	>99/100/>99 ± 2
2	(3)	(4)	98/100/98 ± 3	9	(17)	(18)	52/65/>52 ± 2
3	(5)	(6)	96/100/>96 ± 2	10	(19)	(20)	>99/100/>99 ± 3
4	(7)	(8)	>98/100/>98 ± 3	11	(21)	(22)	>99/100/>99 ± 3
5	(9)	(10)	>99/100/>99 ± 2	12	(23)	(24)	72/100/72 ± 3
6	(11)	(12)	>99/100/>99 ± 2	13	(25)	(26)	>99/100/>99 ± 2
7	(13)	(14)	>99/100/>99 ± 3	14	(27)	(28)	>99/100/>99 ± 3

^a^Unless otherwise stated, 1.0 mmol of the substrate, 1.5 mmol of KOH, and 0.12 mmol% of Aluminum oxy-hydroxide-supported palladium nanoparticles (0.5 wt % Pd) were used under ultrasonic conditions, at 40 °C. ^b1^H NMR conversion based on aromatic substrates. ^c^Selectivity based on ^1^H NMR results. ^d^Determined by ^1^H NMR analysis.

**Figure 5:**
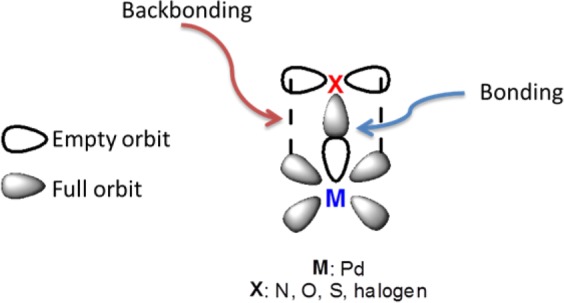
Bonding and back bonding between metal-ligand.

The benzyl alcohol derivatives containing electron-donor groups such as hydroxyl (-OH), methoxy (-OCH_3_), and methyl (-CH_3_) at different positions were also oxidized to the benzaldehyde derivatives in high yields (Table 2, entries 5–8, 13). On the other hand, due to the limitation of motion on the catalyst surface of the molecule with back bonding of (4-(methylthio) phenyl) methanol (17), the reaction efficiency is reduced (Table 2, entry 9). Because the back bonding between the catalyst and the substrate keeps the catalyst away from the reaction center (Fig. 5).

4-nitrobenzaldehyde (20) and 4-(trifluoromethyl) benzaldehyde (28) were obtained with yields of more than 99% (Table 2, entries 10, 14). The benzyl alcohol (21) was oxidized into benzaldehyde (22) with a yield of 99% (Table 2, entry 11). 4-(dimethylamino) benzyl alcohol (23) was converted into 4-(dimethylamino) benzaldehyde (24) with 72% yield (Table 2, entry 12). All trials in Table 2 were repeated at least 3 times.

The oxidation of benzylic alcohols to benzaldehyde derivatives was carried out with high efficiency in the presence of aluminum oxy-hydroxide-supported palladium nanoparticles. Besides its high activity, the aluminum oxy-hydroxide-supported palladium nanoparticles are further stable and reusable for the oxidation reaction, providing ≤ 88% conversion after its 5^th^ consecutive use in the oxidation reaction of various compounds (Table 3). The amount of palladium (~3.5 ppm) infiltrated into the solvent medium after the reaction was determined by ICP-MS. All experiements in Table 3 were performed at least 3 times.

**Table 3:** Recovery test of aluminum oxy-hydroxide-supported palladium nanoparticles.

Entry	Substrate	Product	1st run		5th run	
			Yield^*b*^ (%)	Time, h	Yield^*b*^ (%)	Time, h
1			96 ± 2	3	88 ± 3	3
2			99 ± 3	3	90 ± 2	3

^a^Unless otherwise stated substrate (1.0 mmol), KOH (1.5 mmol) and Aluminum oxy-hydroxide-supported palladium nanoparticles (0.12 mmol%, 0.5 wt.% Pd) were used under ultrasonic conditions at 40 °C. ^b^Determined by 1 H NMR analysis.

**Figure 6:**
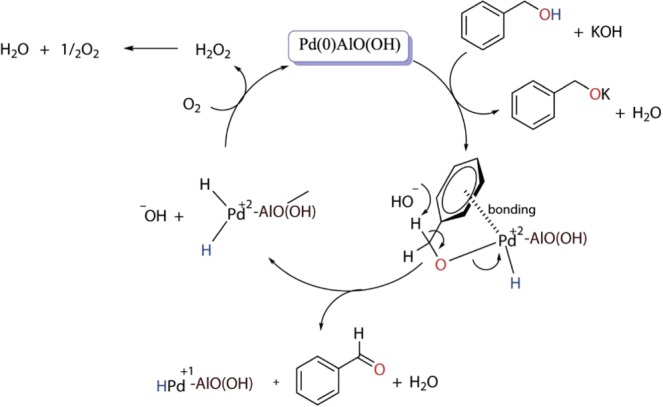
Proposed mechanism for the oxidation of benzyl alcohol.

The proposed reaction mechanism of the oxidation process is represented in Fig. 6. Figure 6 describes the activation of benzyl alcohol in coordination with the first introduction. The coordination of the aromatic ring on the catalyst surface and the coupling of the alcohol oxygen to the metal is the initiating step. Aldehyde formation is then observed by coupling the hydrogen atom in the hydroxyl group with the hydrogen atom in the benzylic position to the catalyst surface. KOH first provides the removal of the alcohol proton and transport it onto the metal. After complex formation, it breaks down the hydrogen in the benzylic position and provides aldehyde formation. Metal (0) is oxidized to metal (+2) in both the coordination and aldehyde formation stages. The M (+2) is again reduced to metal (0) with the presence of molecular oxygen. In this way, the catalyst regains its catalytic activity and ensures the continuity of the oxidation reactions^24^. The catalyst is recovered in the reaction medium as suggested in the mechanism. In addition, because the -OH group in the structure of the catalyst gives the catalyst a basic character, it supports both the transfer of hydrogen of benzylic alcohol and the breakage of the hydrogen in the aldehyde formation step.

## Conclusions

In summary, the results showed that the synthesized aluminum oxy-hydroxide-supported palladium nanoparticles are highly active materials in the conversion of benzylic alcohols to the benzaldehyde derivatives under ultrasonic conditions at 40 °C and in solvent-free media. The products of the aluminum oxy-hydroxide-supported palladium nanoparticles were obtained in the presence of O_2_ within 3 hours, while the recovery efficiencies reached 99%. Moreover, the reaction mechanism behind the oxidation of benzylic alcohols with aluminum oxy-hydroxide-supported palladium nanoparticles was also demonstrated. These results show the key role of the aluminum oxy-hydroxide-supported palladium nanoparticles in the oxidation of alcohols. Of course, since no solvent system is used in the reactions, an environmentally sensitive method has been developed.

## Experimental Section

### Materials

Aluminum oxide-hydroxide supported palladium nanoparticles, benzylic alcohol compounds and KOH used in the experiments were purchased from Sigma-Aldrich. Ultrasonic agitation was carried out using a Laborgerate GmbH 50 Hz 100 W unit.

### General procedure for the oxidation of benzyl alcohol compounds

Into a reaction vessel under ultrasonic conditions and 1 atm of O_2_ were placed aluminum oxy-hydroxide-supported palladium nanoparticles (25 mg, 0.12 mmol%), benzyl alcohol derivatives (1 mmol) and KOH (1.5 mmol). The progress of the reaction was monitored by TLC analysis. After 3 h, the high yield of benzaldehyde compounds was obtained. The products were determined by ^1^H-NMR and ^13^C NMR analysis. The spectral data of the compounds obtained by these analyses are given in the support file.

## Associated Content

TEM images were obtained with a JEOL 200 kV and SEM images were made with a JEOL SEM5800, using Panalitic Empyryl Diffractometer for XRD. Geol ECS 400 MHz spectrometer was used for NMR spectra.

## Supplementary information

Supplementary information.
