# Supplementary material for: Handy and highly efficient oxidation of benzylic alcohols to the benzaldehyde derivatives using heterogeneous Pd/AlO(OH) nanoparticles in solvent-free conditions
Source: Sci Rep. 2020 Mar 31;10:5731. doi: 10.1038/s41598-020-62695-4 (PMC7109032; doi:10.1038/s41598-020-62695-4)
Supplement: Supplementary file 1 — Supplementary information. [file 41598_2020_62695_MOESM1_ESM.docx]

**Supplementary material**

**Handy and highly efficient oxidation of benzylic alcohols to the benzaldehyde derivatives using heterogeneous Pd/AlO(OH) nanoparticles in solvent-free conditions**

**Haydar Goksu*^a^, Fatih Sen*^b^**

*^a^Kaynasli Vocational College, Duzce University, Düzce 81900, Turkey.*

*^b^Sen Research Group, Department of Biochemistry, Dumlupinar University, 43100, Turkey*

**Corresponding Author:** haydargoksu@duzce.edu.tr, fatih.sen@dpu.edu.tr

**Contents (11 pages)**

Page S2 Published works

Page S3 EDX image

Page S4 EDX image

Page S5 XRD pattern

Page S5 TEM images

Page S6 NMR spectra and spectral data for the oxidation products

**Table S1** Comparison of the designed catalytic system with recently published works about oxidation of benzyl alcohols to benzaldehyde

| **Catalyst** | **Conditions** | **Temp.**  ^o^**C** | **Time, h** | **Yield^a^, %** |
| --- | --- | --- | --- | --- |
| CuMn_2_^1^ | Benzyl alcohol (2.0 mmol), catalyst (200 mg), toluene (10ml) | 102 | 1/3 | >99 |
| [VO(TPPABr)] CBr_3_)^2^ | Benzyl alcohol (1.0 mmol), catalyst (5 mg), H_2_O_2_ (1.0 mmol), CH_3_CN (3ml) | 25 | 2 | 90 |
| Au–Cu/SiO_2_^3^ | Benzyl alcohol (0.36 mmol/min), catalyst (0.2 g), O_2_ | 260 | 2 | 98 |
| Co-Bir^4^ | Benzyl alcohol (1.0 mmol), catalyst (50 mg), toluene (10ml), O_2_ | 110 | 24 | 99 |
| CuSO_4_^5^ | Benzyl alcohol (1.0 mmol), catalyst (1 mol%), H_2_O_2_ (1ml, 30%), TEMPO (5 mol%) | 60 | 1 | 99 |
| PdHAP-0^6^ | Benzyl alcohol (1.0 mmol), catalyst (0.1 g), trifluorotoluene (5ml), O_2_ | 90 | 1 | 99 |
| Au–Pd/MgO^7^ | Benzyl alcohol (29.0 mmol), catalyst (0.4 g), water (30ml), O_2_ | 80 | 4 | 52 |
| Au/C^8^ | Benzyl alcohol (1.0 mmol), catalyst (0.3 g), water, H_2_O_2_ (1.25 mmol) | 80 | 6 | 4 |
| Au/Zeolite-Y^8^ | Benzyl alcohol (1.0 mmol), catalyst (0.3 g), water, H_2_O_2_ (1.25 mmol) | 80 | 6 | 94 |
| **PdAlO(OH) NPs (this study)** | Benzyl alcohol (1.0 mmol), catalyst (25 mg), KOH (1.5 mmol), solvent-free, ultrasonic conditions, O_2_ | 25 | 3 | >99 |

^a^isolated yield

1. Ali, R.; Nour, K.; Al-warthan, A.; Siddiqui, M. R. H.; Selective oxidation of benzylic alcohols using copper-manganese mixed oxide nanoparticles as catalyst., Arabian J. Chem. 2015, 8, 512-517; DOI: 10.1016/j.arabjc.2013.05.012.
2. Safaiee, M.; Moeinimehr, M.; Zolfigol, M. A.; Pyridiniumporphyrazinato oxo-vanadium tribromomethanide as a new source of Br+ catalyst for the chemo and homoselective oxidation of sulfides and benzylic alcohols., Polyhedon. 2019, 170, 138-150; DOI: 10.1016/j.poly.2019.05.007.
3. Della Pina, C.; Falletta, E.; Rossi, M.; Highly selective oxidation of benzyl alcohol to benzaldehyde catalyzed by bimetallic gold–copper catalyst., J. Catal. 2008, 260, 384–386; DOI:10.1016/J.JCAT.2008.10.003.
4. Kamimura, A.; Nozaki, Y.; Nishiyama, M.; Nakayama, M.; Oxidation of benzyl alcohols by semi-stoichiometric amounts of cobalt-doped birnessite-type layered MnO2 under oxygen atmosphere., RSC Adv. 2013, 3, 468–472; DOI:10.1039/c2ra22117a.
5. Ahmad, J. U.; Raisanen, M. T.; Leskela, M.; Repo, T.; Copper catalyzed oxidation of benzylic alcohols in water with H_2_O_2_., Appl. Catal. A. 2012, 411-412, 180-187; DOI: 10.1016/j.apcata.2011.10.038.
6. Mori, K.; Hara, T.; Mizugaki, T.; Ebitani, K.; Kaneda, K.; Hydroxyapatite-supported palladium nanoclusters:  A highly active heterogeneous catalyst for selective oxidation of alcohols by use of molecular oxygen., J. Am. Chem. Soc. 2004, 126, 10657–10666; DOI:10.1021/ja0488683.
7. Zhan, G.; Hong, Y.; Mbah, V. T.; Huang, J.; Ibrahim, A.-R.; Du, M.; Li, Q.; Bimetallic Au–Pd/MgO as efficient catalysts for aerobic oxidation of benzyl alcohol: A green bio-reducing preparation method., Appl. Catal. A. 2012, 439-440, 179-186; DOI: [10.1016/j.apcata.2012.07.005](https://doi.org/10.1016/j.apcata.2012.07.005).
8. Zhan, G.; Huang, J.; Du, M.; Sun, D.; Ibrahim, A.-R.; Lin, W.; Hong, Y.; Li, Q.; Liquid phase oxidation of benzyl alcohol to benzaldehyde with novel uncalcined bioreduction Au catalysts: High activity and durability., Chem. Eng. J. 2012, 187, 232-238; DOI: [10.1016/j.cej.2012.01.051](https://doi.org/10.1016/j.cej.2012.01.051)

**
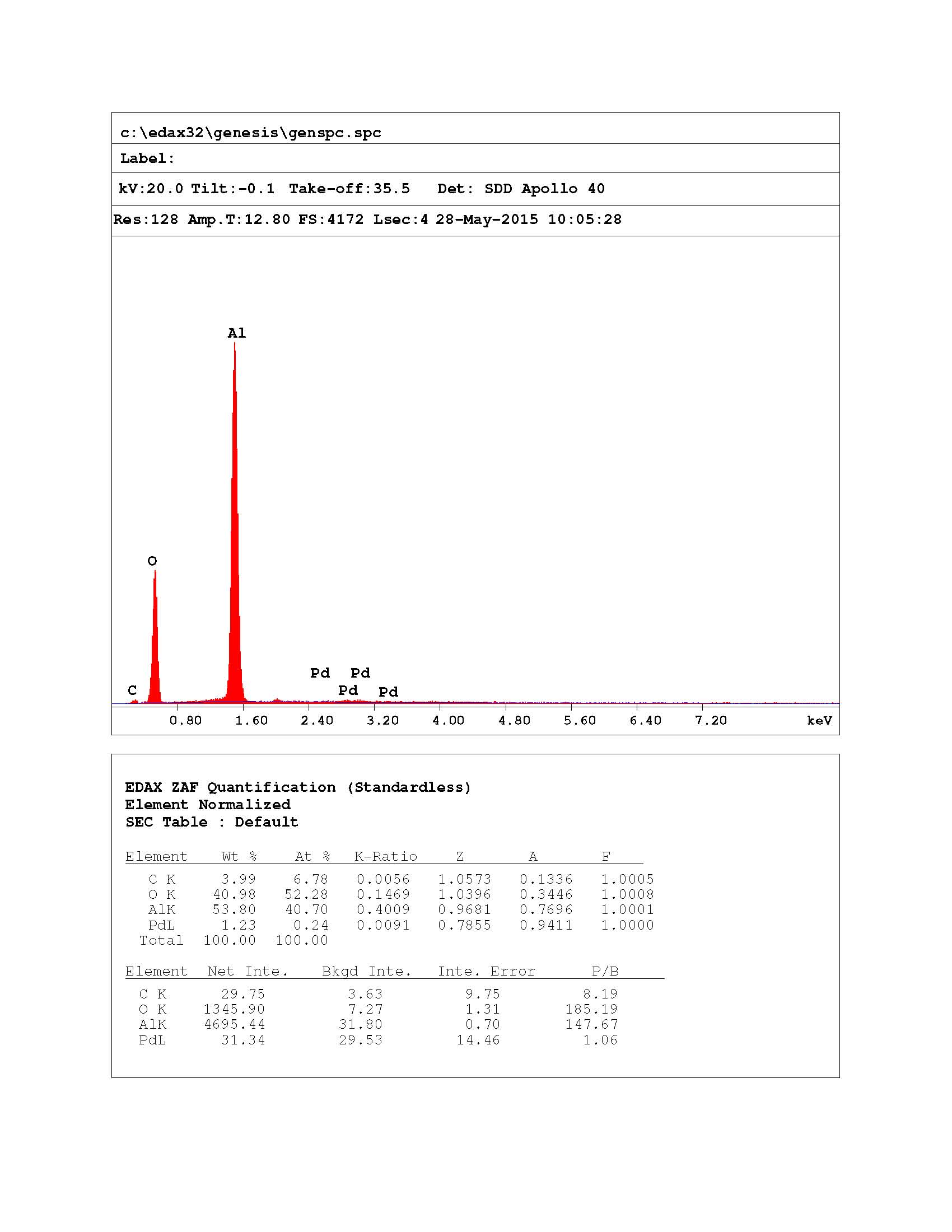
**

**Figure S1** EDX spectrum before the reaction of Pd/AlO(OH) NPs.

**
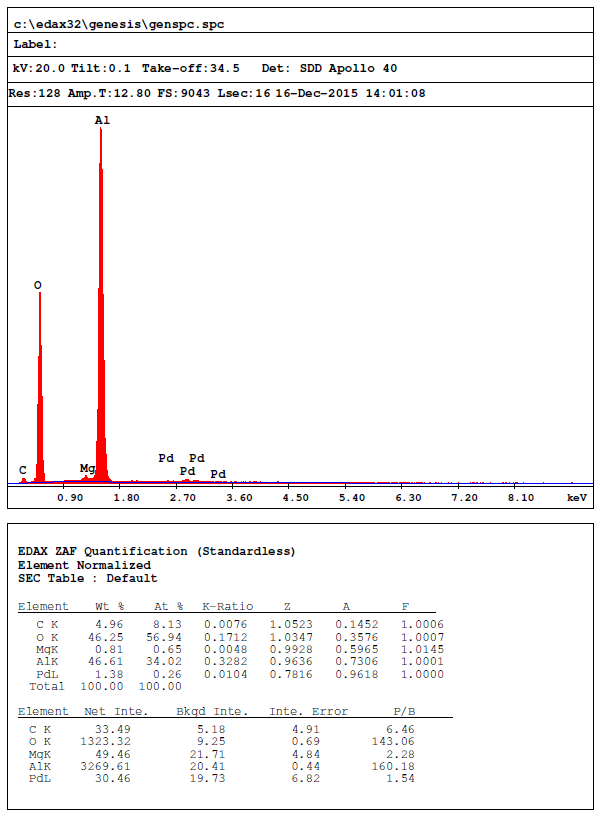
**

**Figure S2** EDX spectrum after using five times of Pd/AlO(OH) NPs.


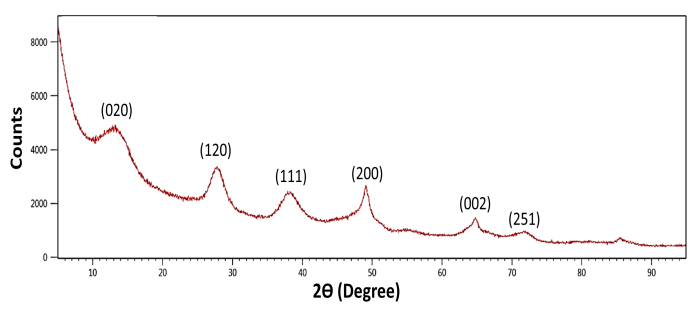


**Figure S3** XRD pattern spectrum of Pd/AlO(OH).

**
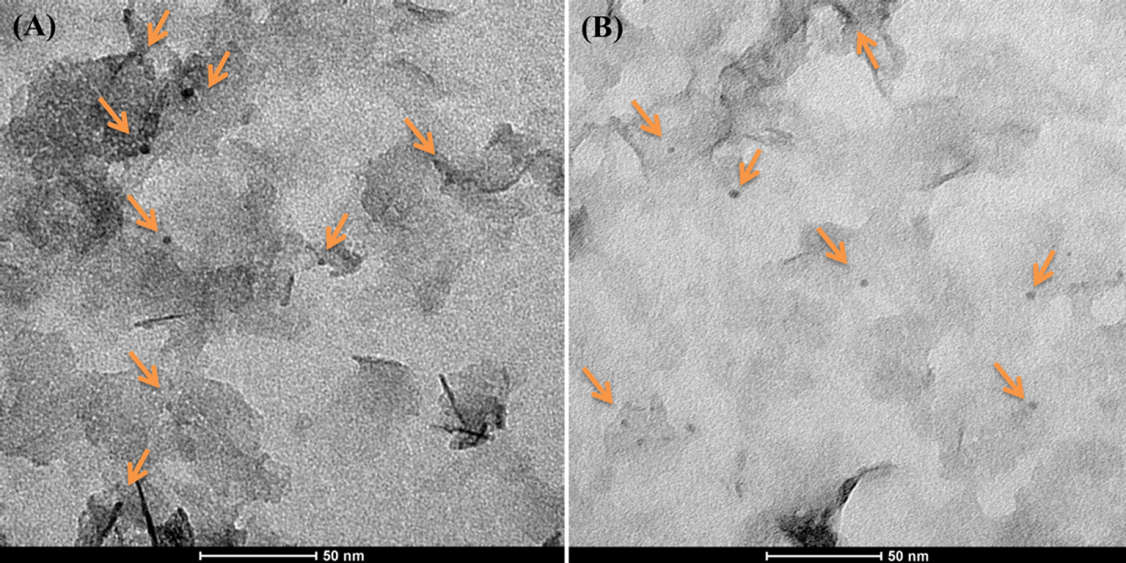
**

**Figure S4** TEM images: (**A)** Pd/AlO(OH) NPs before the reaction; (**B)** Pd/AlO(OH) NPs after reusing five times

**^1^H-NMR Spectra for Oxidation Products**

**Spectral data of compounds**

*2-fluorobenzaldehyde* ***(2)****:* ^1^H NMR (400 MHz, CDCl_3_): *δ* 10.36 (s, 1H), 7.92-7.79 (m, 1H), 7.68-7.52 (m, 1H), 7.34-7.21 (m, 1H), 7.20-7.08 (m, 1H). ^13^C NMR (100 MHz, CDCl_3_): *δ* 187.4, 166.1, 136.5, 128.8, 124.7, 124.2, 116.7.

*4-fluorobenzaldehyde* ***(4):*** ^1^H NMR (400 MHz, CDCl_3_): *δ* 9.94 (s, 1H), 7.95-7.80 (m, 2H), 7.29-7.12 (m, 2H), 7.99 (d, *J* = 8.1 Hz, 2H), 7.79 (d, *J* = 8.1 Hz, 2H). ^13^C NMR (100 MHz, CDCl_3_): *δ* 190.6, 167.9, 165.3, 132.4, 132.3, 116.5, 116.3.

*4-bromobenzaldehyde* ***(6):*** ^1^H NMR (400 MHz, CDCl_3_): *δ* 9.96 (s, 1H), 7.77-7.71 (m, 2H), 7.70-7.64 (m, 2H). ^13^C NMR (100 MHz, CDCl_3_): *δ* 191.2, 131.9, 131.7, 131.1, 129.9.

*3,4-dichlorobenzaldehyde* ***(8):*** ^1^H NMR (400 MHz, CDCl_3_): *δ* 9.94 (s, 1H), 8.15 (d, *J* = 2.0 Hz, 1H), 7.89 (dd, *J* = 8.4, 2.0 Hz, 1H), 7.53 (m, 1H). ^13^C NMR (100 MHz, CDCl_3_): *δ* 192.9, 133.8, 132.1, 131.9, 126.4.

*4-hydroxybenzaldehyde* ***(10):*** ^1^H NMR (400 MHz, CDCl_3_): *δ* 9.85 (s, 1H), 7.88-7.70 (m, 2H), 7.04-6.86 (m, 2H). ^13^C NMR (100 MHz, CDCl_3_): *δ* 191.3, 161.6, 132.6, 127.5, 116.1.

*4-methoxybenzaldehyde* ***(12):*** ^1^H NMR (400 MHz, CDCl_3_): *δ* 9.88 (s, 1H), 7.83 (d, *J* = 8.6 Hz, 2H), 7.00 (d, *J* = 8.6 Hz, 2H), 3.88 (s, 3H). ^13^C NMR (100 MHz, CDCl_3_): *δ* 190.5, 132.1, 111.5, 40.4.

*2,5-dimethoxybenzaldehyde* ***(14):*** ^1^H NMR (400 MHz, CDCl_3_): *δ* 10.43 (s, 1H), 7.31 (d, *J* = 3.3 Hz, 1H), 7.17-7.07 (m, 1H), 6.93 (d, *J* = 9.1 Hz, 1H), 3.88 (s, 3H), 3.78 (s, 3H). ^13^C NMR (100 MHz, CDCl_3_): *δ* 189.7, 156.8, 153.6, 123.6, 113.4, 110.4, 56.2, 55.9.

*3,4,5-trimethoxybenzaldehyde* ***(16):*** ^1^H NMR (400 MHz, CDCl_3_): *δ* 9.85 (s, 1H), 7.11 (s, 2H), 3.92 (s, 9 H). ^13^C NMR (100 MHz, CDCl_3_): *δ* 191.2, 153.7, 143.9, 131.8, 106.7, 61.1, 56.3.

*4-nitrobenzaldehyde* ***(20):*** ^1^H NMR (400 MHz, CDCl_3_): *δ* 10.15 (s, 1H), 8.51-8.29 (m, 2H), 8.20-7.91 (m, 2H). ^13^C NMR (100 MHz, CDCl_3_): *δ* 190.4, 140.1, 130.6, 124.4.

*Benzaldehyde* ***(22):*** ^1^H NMR (400 MHz, CDCl_3_): *δ* 9.99 (s, 1H), 7.99 (dd, *J* = 5.9, 2.0 Hz, 2H), 7.64-7.56 (m, 1H), 7.54-7.45 (m, 2H). ^13^C NMR (100 MHz, CDCl_3_): *δ* 192.5, 136.4, 134.6, 129.8, 129.1.

*4-(dimethylamino)benzaldehyde* ***(24):*** ^1^H NMR (400 MHz, CDCl_3_): *δ* 9.76 (s, 1H), 7.78-7.70 (m, 2H), 6.74 (d, *J* = 8.9 Hz, 2H), 3.08 (s, 6H). ^13^C NMR (100 MHz, CDCl_3_): *δ* 190.5, 132.1, 111.5, 40.4.

*4-methylbenzaldehyde* ***(26):*** ^1^H NMR (400 MHz, CDCl_3_): *δ* 9.95 (s, 1H), 7.76 (d, *J* = 8.0 Hz, 2H), 7.32 (d, *J* = 8.0 Hz, 2H), 2.42 (s, 3H). ^13^C NMR (100 MHz, CDCl_3_): *δ* 192.2, 145.7, 134.2, 129.9, 129.8, 21.9.

*4-(trifluoromethyl)benzaldehyde* ***(28):*** ^1^H NMR (400 MHz, CDCl_3_): *δ* 10.08 (s, 1H), 7.99 (d, *J* = 8.1 Hz, 2H), 7.79 (d, *J* = 8.1 Hz, 2H). ^13^C NMR (100 MHz, CDCl_3_): *δ* 191.2, 130.0, 126.2, 126.1.

**2-fluorobenzaldehyde:** ^1^H NMR (400 MHz, CDCl_3_): *δ* 10.36 (s, 1H), 7.92-7.79 (m, 1H), 7.68-7.52 (m, 1H), 7.34-7.21 (m, 1H), 7.20-7.08 (m, 1H). ^13^C NMR (100 MHz, CDCl_3_): *δ* 187.4, 166.1, 136.5, 128.8, 124.7, 124.2, 116.7.

**4-fluorobenzaldehyde:** ^1^H NMR (400 MHz, CDCl_3_): *δ* 9.94 (s, 1H), 7.95-7.80 (m, 2H), 7.29-7.12 (m, 2H), 7.99 (d, *J* = 8.1 Hz, 2H), 7.79 (d, *J* = 8.1 Hz, 2H). ^13^C NMR (100 MHz, CDCl_3_): *δ* 190.6, 167.9, 165.3, 132.4, 132.3, 116.5, 116.3.

**4-bromobenzaldehyde:** ^1^H NMR (400 MHz, CDCl_3_): *δ* 9.96 (s, 1H), 7.77-7.71 (m, 2H), 7.70-7.64 (m, 2H). ^13^C NMR (100 MHz, CDCl_3_): *δ* 191.2, 131.9, 131.7, 131.1, 129.9.

**
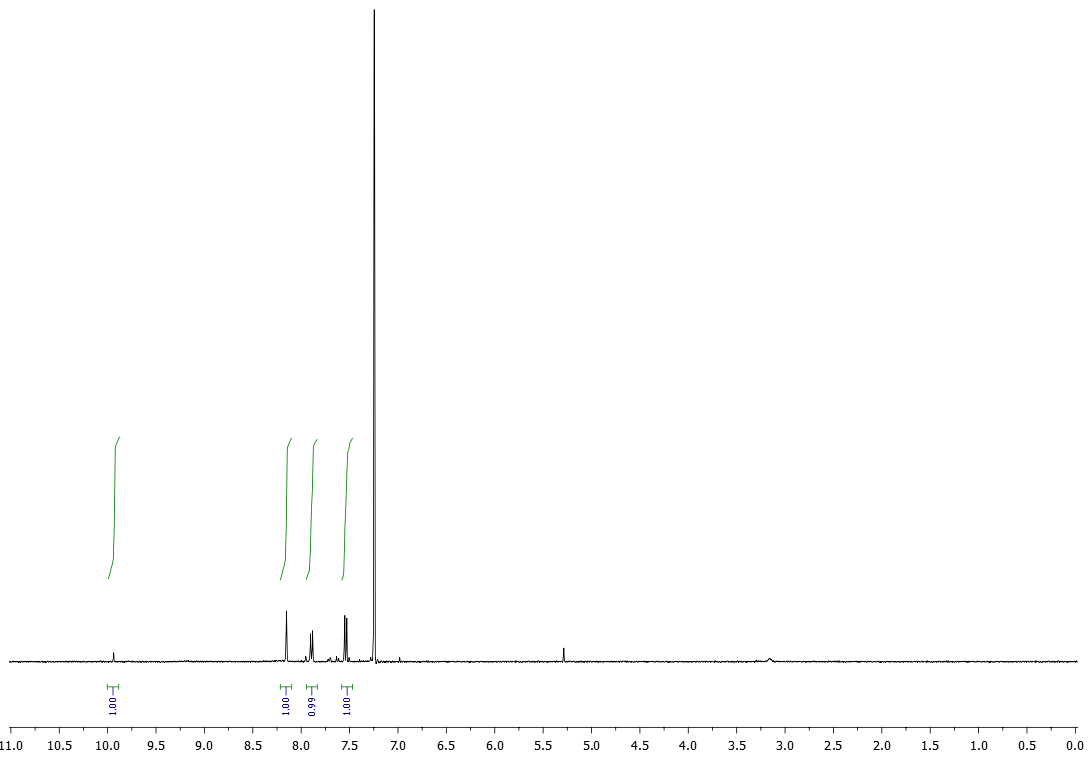
**

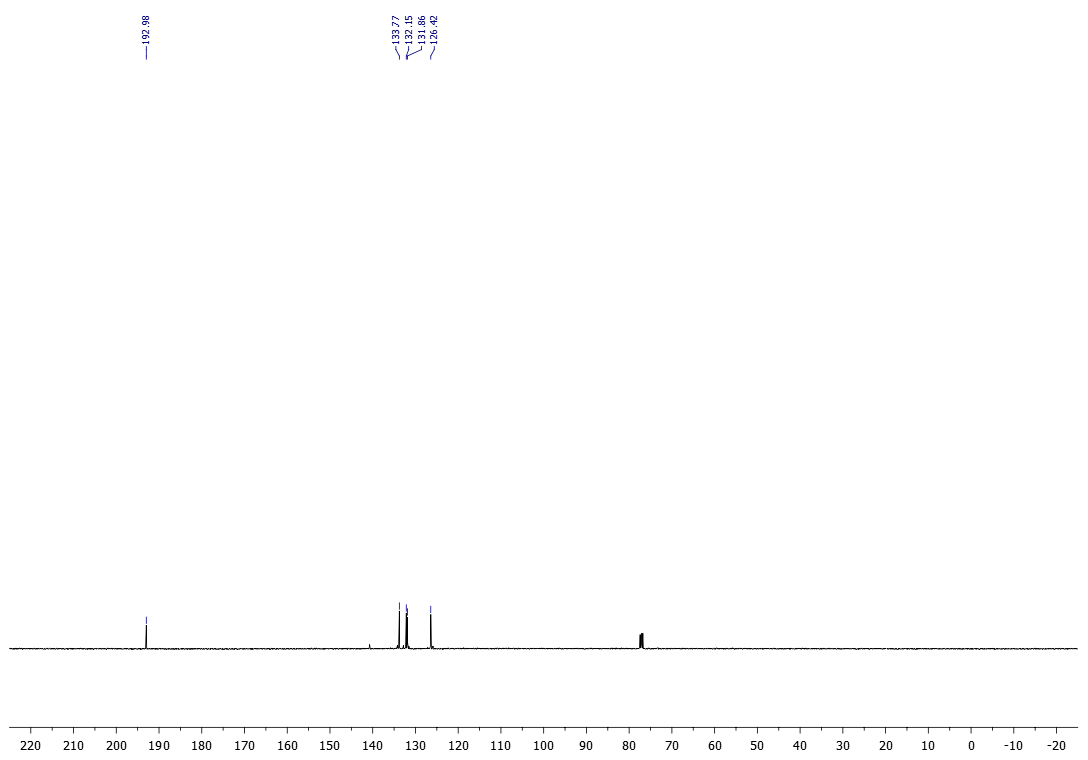


**3,4-dichlorobenzaldehyde:** ^1^H NMR (400 MHz, CDCl_3_): *δ* 9.94 (s, 1H), 8.15 (d, *J* = 2.0 Hz, 1H), 7.89 (dd, *J* = 8.4, 2.0 Hz, 1H), 7.53 (m, 1H). ^13^C NMR (100 MHz, CDCl_3_): *δ* 192.9, 133.8, 132.1, 131.9, 126.4.

**4-hydroxybenzaldehyde:** ^1^H NMR (400 MHz, CDCl_3_): *δ* 9.85 (s, 1H), 7.88-7.70 (m, 2H), 7.04-6.86 (m, 2H). ^13^C NMR (100 MHz, CDCl_3_): *δ* 191.3, 161.6, 132.6, 127.5, 116.1.

**
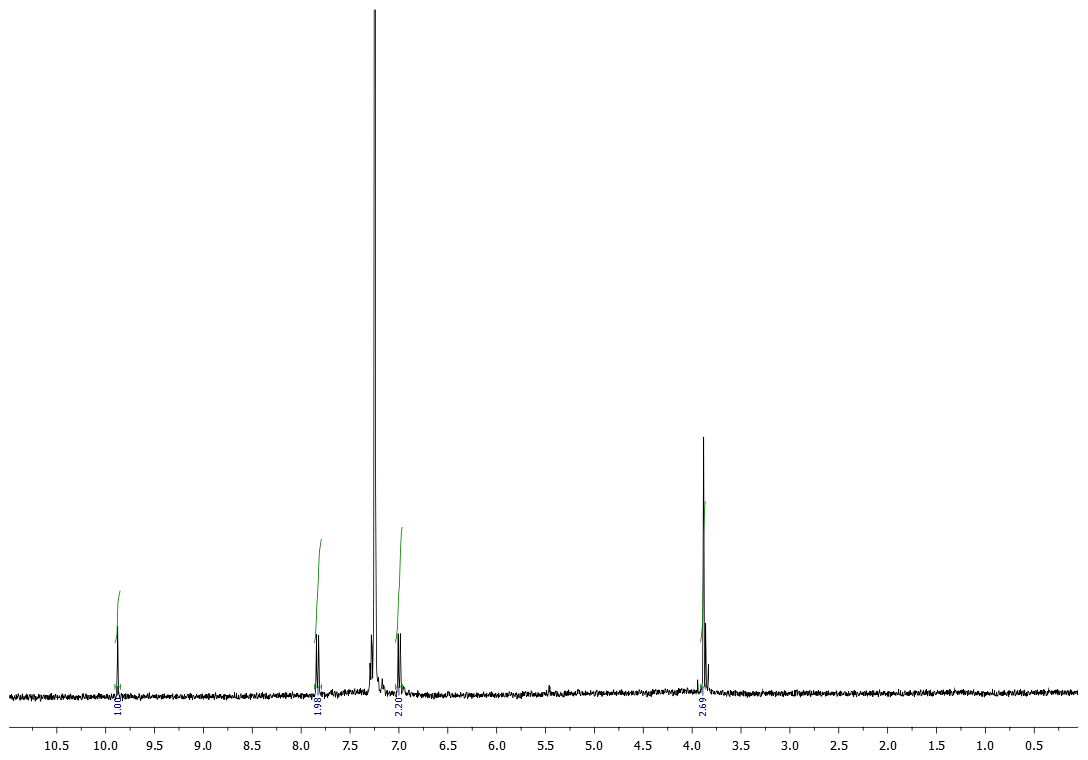
**

**4-methoxybenzaldehyde:** ^1^H NMR (400 MHz, CDCl_3_): *δ* 9.88 (s, 1H), 7.83 (d, *J* = 8.6 Hz, 2H), 7.00 (d, *J* = 8.6 Hz, 2H), 3.88 (s, 3H). ^13^C NMR (100 MHz, CDCl_3_): *δ* 190.5, 132.1, 111.5, 40.4.

**2,5-dimethoxybenzaldehyde:** ^1^H NMR (400 MHz, CDCl_3_): *δ* 10.43 (s, 1H), 7.31 (d, *J* = 3.3 Hz, 1H), 7.17-7.07 (m, 1H), 6.93 (d, *J* = 9.1 Hz, 1H), 3.88 (s, 3H), 3.78 (s, 3H). ^13^C NMR (100 MHz, CDCl_3_): *δ* 189.7, 156.8, 153.6, 123.6, 113.4, 110.4, 56.2, 55.9.

**3,4,5-trimethoxybenzaldehyde:** ^1^H NMR (400 MHz, CDCl_3_): *δ* 9.85 (s, 1H), 7.11 (s, 2H), 3.92 (s, 9 H). ^13^C NMR (100 MHz, CDCl_3_): *δ* 191.2, 153.7, 143.9, 131.8, 106.7, 61.1, 56.3.


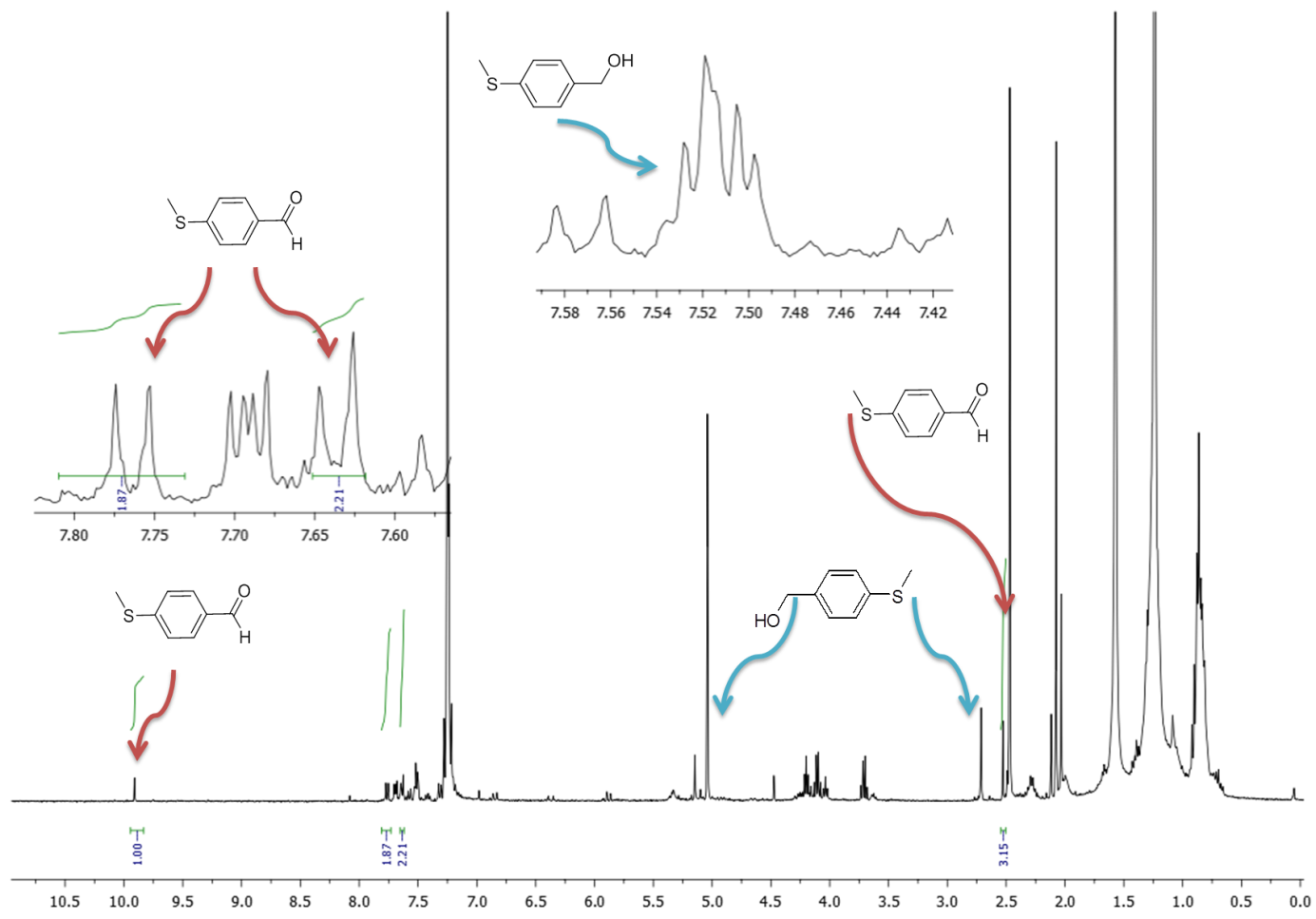


**4-(methylthio)benzaldehyde**

**4-nitrobenzaldehyde:** ^1^H NMR (400 MHz, CDCl_3_): *δ* 10.15 (s, 1H), 8.51-8.29 (m, 2H), 8.20-7.91 (m, 2H). ^13^C NMR (100 MHz, CDCl_3_): *δ* 190.4, 140.1, 130.6, 124.4.

**Benzaldehyde:** ^1^H NMR (400 MHz, CDCl_3_): *δ* 9.99 (s, 1H), 7.99 (dd, *J* = 5.9, 2.0 Hz, 2H), 7.64-7.56 (m, 1H), 7.54-7.45 (m, 2H). ^13^C NMR (100 MHz, CDCl_3_): *δ* 192.5, 136.4, 134.6, 129.8, 129.1.

**4-(dimethylamino)benzaldehyde:** ^1^H NMR (400 MHz, CDCl_3_): *δ* 9.76 (s, 1H), 7.78-7.70 (m, 2H), 6.74 (d, *J* = 8.9 Hz, 2H), 3.08 (s, 6H). ^13^C NMR (100 MHz, CDCl_3_): *δ* 190.5, 132.1, 111.5, 40.4.

**4-methylbenzaldehyde:** ^1^H NMR (400 MHz, CDCl_3_): *δ* 9.95 (s, 1H), 7.76 (d, *J* = 8.0 Hz, 2H), 7.32 (d, *J* = 8.0 Hz, 2H), 2.42 (s, 3H). ^13^C NMR (100 MHz, CDCl_3_): *δ* 192.2, 145.7, 134.2, 129.9, 129.8, 21.9.

**4-(trifluoromethyl)benzaldehyde:** ^1^H NMR (400 MHz, CDCl_3_): *δ* 10.08 (s, 1H), 7.99 (d, *J* = 8.1 Hz, 2H), 7.79 (d, *J* = 8.1 Hz, 2H). ^13^C NMR (100 MHz, CDCl_3_): *δ* 191.2, 130.0, 126.2, 126.1.
